# Major explosions and paroxysms at Stromboli (Italy): a new historical catalog and temporal models of occurrence with uncertainty quantification

**DOI:** 10.1038/s41598-020-74301-8

**Published:** 2020-10-15

**Authors:** Andrea Bevilacqua, Antonella Bertagnini, Massimo Pompilio, Patrizia Landi, Paola Del Carlo, Alessio Di Roberto, Willy Aspinall, Augusto Neri

**Affiliations:** 1 Istituto Nazionale di Geofisica e Vulcanologia, Sezione di Pisa, Pisa, Italy; 2 School of Earth Sciences, University of Bristol, Bristol, UK

**Keywords:** Natural hazards, Volcanology

## Abstract

Stromboli volcano (Italy), always active with low energy explosive activity, is a very attractive place for visitors, scientists, and inhabitants of the island. Nevertheless, occasional more intense eruptions can present a serious danger. This study focuses on the modeling and estimation of their inter-event time and temporal rate. With this aim we constructed a new historical catalog of major explosions and paroxysms through a detailed review of scientific literature of the last ca. 140 years. The catalog includes the calendar date and phenomena descriptions for 180 explosive events, of which 36 were paroxysms. We evaluated the impact of the main sources of uncertainty affecting the historical catalog. In particular, we categorized as uncertain 45 major explosions that reportedly occurred before 1985 and tested the effect of excluding these events from our analysis. Moreover, after analyzing the entire record in the period [1879, 2020], we separately considered, as sequences, events in [1879, 1960] and in [1985, 2020] because of possible under recording issues in the period [1960, 1985]. Our new models quantify the temporal rate of major explosions and paroxysms as a function of time passed since the last event occurred. Recurrence hazard levels are found to be significantly elevated in the weeks and months following a major explosion or paroxysm, and then gradually decrease over longer periods. Computed hazard functions are also used to illustrate a methodology for estimating order-of-magnitude individual risk of fatality under certain basis conditions. This study represents a first quantitatively formal advance in determining long-term hazard levels at Stromboli.

## Introduction

Stromboli volcano (Italy) represents a great tourist attraction, thanks to the daily, mild Strombolian explosions that characterize its ordinary activity. Stromboli is also considered a unique “laboratory volcano” attracting many scientists each year for volcanological investigations and monitoring experiments. Nevertheless, occasional more intense eruptions can present a danger to visitors and inhabitants. How to keep people safe from these sudden, larger eruptions is a challenging task. Our purpose is to obtain, for the first time, a detailed assessment of the timings of such violent explosions, thus providing information and an objective basis to develop quantitative hazard and risk assessments.

**Figure 1:**
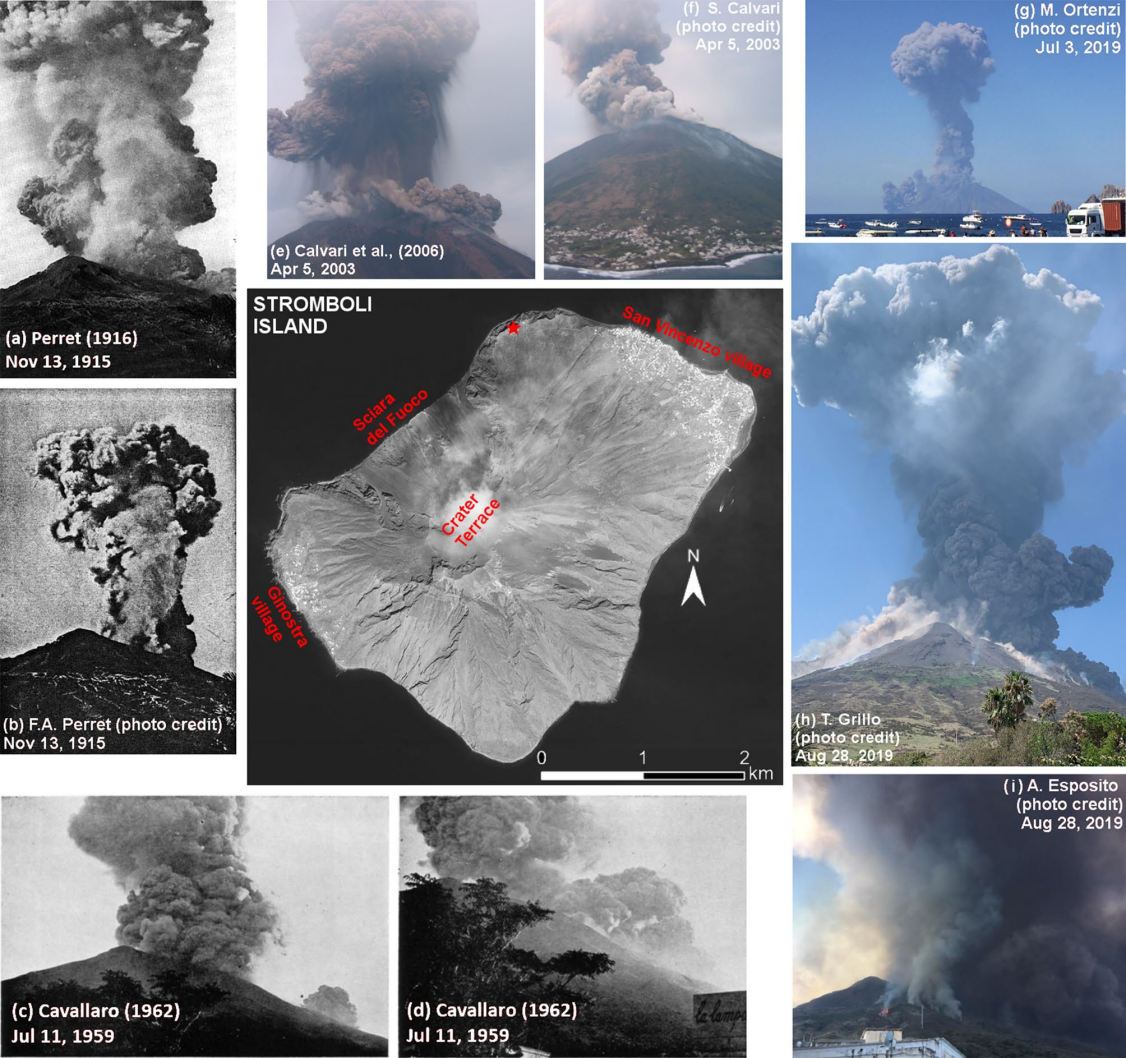
In the middle, we show an aerial overview of the island modified from Bertagnini et al.^37^. The red star marks the location of Labronzo lighthouse. In (**a**–**i**) we show historical photographs of five paroxysms, from historical sources^49,52,96^, and credited photographs. (**e**,**f**) Photos of S. Calvari, (**g**) is a photo of M. Ortenzi, (**h**) is a photo of T. Grillo, and (**i**) is a photo of A. Esposito, published with their permission.

The Island of Stromboli is the 3 × 4 km visible portion of a much larger stratovolcano, with a landmass slightly elongated in the NE-SW direction, which extends to a depth of 1500–2600 m below sea level. Only one third of the volcanic edifice emerges above sea level, reaching an elevation of 924 m. The island is characterized by the presence of a horseshoe-shaped depression called Sciara del Fuoco in the NW sector of the volcano, which is 1700 m wide, and extends underwater to a depth over 2000 m. The current volcanic activity takes place in several craters located within a flat area called Crater Terrace, at about 750 m of elevation on top of Sciara del Fuoco (Fig. 1). Typically, activity is Strombolian, characterized by continuous degassing accompanied with episodic, mild to moderate explosions lasting a few seconds, and ejecting incandescent scoriaceous lapilli and bombs, ash, and lithic blocks, capable of reaching 100–200 m height above the craters^79,94,123,124^. Different eruptive vents can be the source of the explosions, and they often change in shape and number. Strombolian explosions have variable inter-event times, ranging from minutes to hours, with an average inter-event gap of 10–20 min. Sometimes, lava flows originate from the summit craters or from eruptive fractures within the Sciara del Fuoco^28,48,113,118^.

Occasionally, sudden, more violent explosions named “Strombolian paroxysms”^83^ interrupt the ordinary activity of Stromboli. During these explosions, several craters can be almost simultaneously involved, erupting volumes of materials much larger and with a considerably higher energy than during ordinary activity. These violent explosions range from small-scale paroxysms^37,118^, also called “major explosions”^28^, to large-scale paroxysms, for simplicity defined “paroxysms”^28,38,118^. Figures 1a–i show some historical examples of this type of phenomena. During major explosions, up to m-sized ejecta, including both lithic blocks and juvenile scoriaceous bombs, typically affect the summit part of the volcano, up to a maximum distance of about 1 km from the vents, where they can ignite fires. Short-living ash plumes may rise up to about 1 km above the craters, and ash fallout can affect the whole downwind portion of the island^118^. These episodes typically relate to activity from multiple vents and can include sequences of explosions separated by a few minutes^9,35,50,70,72^. Strong detonations may be heard on the island and even shock waves capable of shaking buildings can be common during major explosions^20,59,90,107^.

The paroxysms have been the most violent eruptive manifestations at Stromboli since the beginning of the present style of activity, in the VIII century AD^38,116,118^. These are highly energetic multiple explosions lasting a few minutes and ejecting up to meter-sized pumiceous/scoriaceous bombs and lithic blocks at distances up to 2.5 km from the vents. They are accompanied by the formation of a convective ash-gas plume that rises 3–4 km above the volcano (up to 10 km in the most large-scale events, e.g. May 22 1919^103^), bearing ash, scoria and lapilli^10,36,37,49,88,97,98,117^. During the paroxysms, shock waves can break window glass across the island, with detonations heard tens of km away in Calabria and Sicily^3,103,110^. Pyroclastic density currents (PDC), usually confined within the Sciara del Fuoco, are often associated with paroxysms. In a few cases, hot avalanches generated by remobilization of pyroclastic material can spread outside Sciara del Fuoco^96,104,106,107^. For instance, in the paroxysm of September 11, 1930, hot avalanches reached Stromboli village causing severe damage to some buildings and killing at least four people^64,89,115,122^. PDCs and hot avalanches entering the sea can generate small tsunamis affecting the island^2,51,83,102^.

There is some evidence that a continuous increase of energy characterizes such more intense explosive activity, particularly in the transition from the ordinary activity and the major explosions^8,9,114^. In this study, we maintain the common classification into two categories: “paroxysm” or “major explosion”. We remark that, while a measure of energy is unavailable for the historical events, the above classification is not, a priori, in contrast with a continuous increase of energy. Thus, the two categories of major explosions and paroxysms are approximately equivalent to cut the energetic scale above and below specific thresholds.

This study performs statistical modeling of inter-event times and temporal rates of the violent explosive phenomena of Stromboli volcano, i.e. paroxysms as a category, and major explosions and paroxysms taken together (ME&P). We based our analysis on a new catalog of historical major explosions and paroxysms from the last ca. 140 years, which we compiled for this specific purpose. In particular: (1) we tested classical renewal models, and (2) we developed a new model with two different probability assessments for short and long inter-event times, corresponding to a two-state hidden Markov chain^25,32^. Both these approaches are capable of replicating the temporal pattern of ME&P better than a Poisson model and naturally reproduce clusters of events^33,40,44^. In the analysis we also considered the two main sources of epistemic uncertainty affecting the record of ME&P: (1) under-recording issues, and (2) uncertain distinction of a significant number of major explosions from ordinary activity. We represent some probability estimates using ill-constrained information and we describe them with a range of values^39,129^.

The study is structured in two main parts: the first focuses on the quantitative description of the past record of ME&P, while the second part details the forecasting models. The models allow us to quantify the temporal rate of ME&P as a function of time since the last event occurred, i.e. as hazard functions^56,57^. In order to highlight the relevance of such hazard functions for risk assessment, we also estimated the equivalent time to occurrence of an ME&P event with probability P, where P = 0.1% or P = 0.01%. This allows us to estimate also, to an order-of-magnitude, the total annual tolerable amount of time that individuals (e.g. volcanologists, guides, and tourists) could spend in the summit zones most exposed to the related hazards^61,77^. We remark that our estimates are illustrative of the methodology and based only on the individual risk of fatality, since we do not assess societal risk or economical loss^71,73,120^. Moreover, we do not consider the spatial component of risk exposure and assume an exposed person is lethally vulnerable. Thus the presented risk values are maximal for fatality—i.e. they will decrease with distance from the crater area and could be mitigated in a number of ways—which are not taken into account here.

## Historical record of major explosions and paroxysms at Stromboli

Our statistical analysis relies on a new historical record of 180 ME&P events that occurred in the period [1879, 2020]. In particular, we updated and expanded the historical record of Barberi et al.^28^ and incorporated the [1990, 2011] record of Rosi et al.^118^. The new dataset contains the calendar day of the ME&P, and the event distinction between major explosion or paroxysm, plus additional information on the main phenomena observed. The historical record is up-to-date as of 31/8/2020.

We used data from the [1879, 1985] historical catalog of Bevilacqua et al.^47^. The catalog includes the ME&P record and a sequence of extracts from the original literature sources before 1985 that helped us in the complex characterization of the less recent events. We summarized the event information content in five columns—(1) noise and earthquakes, (2) ash plume, (3) large ejecta, (4) PDC and tsunami, (5) lava flows. Where possible, the extracts contained the description of any volcanic activity at least 1 month before the eruption and 1 month after.

In our analysis, we mostly relied on the detailed characterization of ME&P, and paroxysms, in Rosi et al.^118^ that provided quantitative constraints on total duration, fallout volume, mass discharge rate, ballistic size, ballistic range and column height of ordinary activity, major explosions, and paroxysms. In several cases, we had to carefully evaluate the original description of the phenomena, due to insufficient quantitative information in the scientific literature. In particular, we considered the area affected by large ballistic projectiles as the discriminant factor to distinguish between ordinary activity, major explosions and paroxysms. As mentioned above, this area is limited to the Crater Terrace (Fig. 1) in case of ordinary activity, to the summit area of the volcano and Sciara del Fuoco during major explosions, and can extend down to low elevations along large part of the island, and sometimes beyond the shoreline, during the paroxysms^28^. We also considered several other factors, including the height of the plume, the amount of ash and scoria fallout, the occurrence and strength of any associated shock wave. The occurrence of PDC and/or tsunami associated with the violent explosive activity was assumed as a marker for a paroxysm too^118^. The determination of the hour and minute of the explosion in the historical documents usually distinguishes the most violent phenomena. Some events were atypical and required further assessment. For instance, we considered the eruption of July–August 1912 a paroxysm^95^, although the violent activity lasted for several days without the observation of a clearly climactic event, because exceptional (for Stromboli) deposits of two meters on the Crater Terrace characterized that eruption. Another atypical eruption that we considered a paroxysm occurred on November 18, 1882^84^. In that case five temporary vents opened 100 m downslope in Sciara del Fuoco instead of on the Crater Terrace. The violence of the explosion and the volume and size of ejecta drove its characterization as a paroxysm.

**Figure 2:**
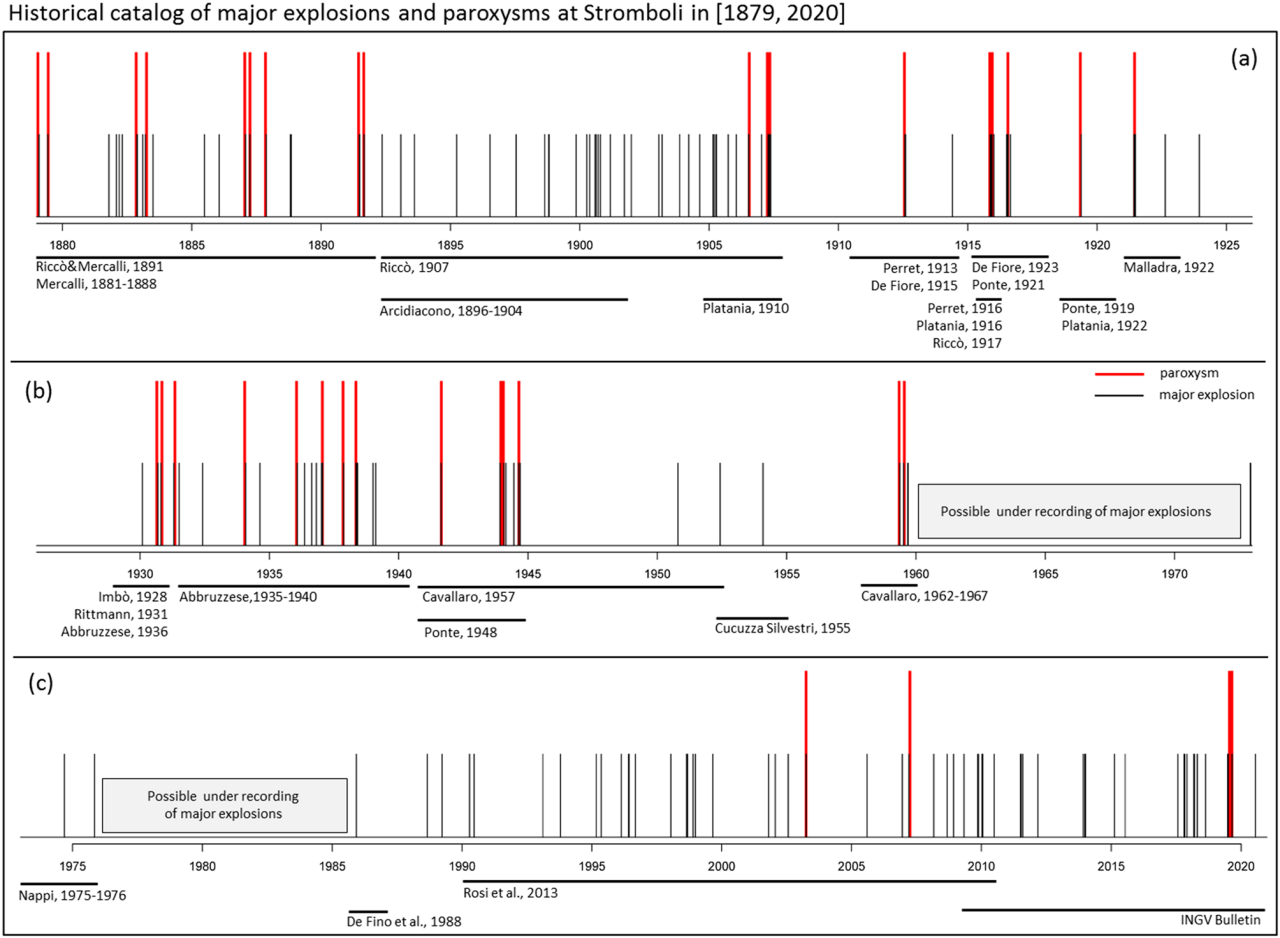
Historical catalog of ME&P at Stromboli in [1879, 2020]. (**a**) [1879, 1926], (**b**) [1926, 1973], (**c**) [1973, 2020]. Black bars mark the major explosions and red bars mark the paroxysms. We show the time intervals described in the key literature sources below the barplot. The key sources are: Mercalli^83–87^; Riccò and Mercalli^110^; Arcidiacono^12–23^; Riccò^107,109^; Platania^100–102^; Perret^95,96^; De Fiore^59,60^; Ponte^103–106^; Malladra^81^; Imbò^74^; Rittmann^115^; Abbruzzese^1–4^; Cucuzza Silvestri^55^; Cavallaro^51–53^; Nappi^90,91^; De Fino et al.^58^; Rosi et al.^118^ and the INGV periodic bulletin of Stromboli (https://www.ct.ingv.it).

Whilst identifying the paroxysms was relatively straightforward, several possible major explosions were not clearly distinguishable from particularly violent episodes of the ordinary Strombolian activity. Thus, our historical record includes the quantification of the main sources of uncertainty, i.e. the possibility of major explosions of uncertain characterization because of insufficient information, and of the possible periods of under-recording^39,44,125^, as described in the sequel. The full [1879, 2020] record of Stromboli ME&P is displayed in Fig. 2a–c and is available as Supporting Information S1.

In addition to direct observations made by people living on the island, and to the scientific literature studying the topic, the information collected by military personnel on duty at the lighthouse of Stromboli greatly improved the completeness of the historical record of ME&P and deserves special attention^85,108^. The Labronzo lighthouse was located 200 m to the East of Sciara del Fuoco, in sight of the craters (Fig. 1). Informally, the scientific observations made by the military personnel of the lighthouse started before 1890. A more detailed monitoring service became institutional in 1898 after an agreement between the Observatory of Catania and the Italian Navy^107,110^. The Great Calabrian Earthquake of 1905 severely damaged the lighthouse, and the military personnel moved to a temporary edifice out of sight of the craters until 1915. However, the reports continued, thanks to frequent and regular scouting hikes^95,109^. Then, the old lighthouse building was garrisoned in 1916, but unoccupied in 1919^102,104^. A large ballistic projectile destroyed the building in 1930^2,74,115^. The daily observations made by the military continued until 1939, when the government suspended them due to lack of personnel. This monitoring service was restored and operative again between 1953 and 1958 and then definitely interrupted^51,53^.

After the end of the military observations, significant under-recording issues might affect the record of major eruptions, until 1985 and the beginning of automated monitoring carried out by INGV network. However, it is unlikely that the island population would have failed to report any paroxysm that occurred after 1959.

## Recurrence rate of ME&P and of paroxysms only

**Figure 3:**
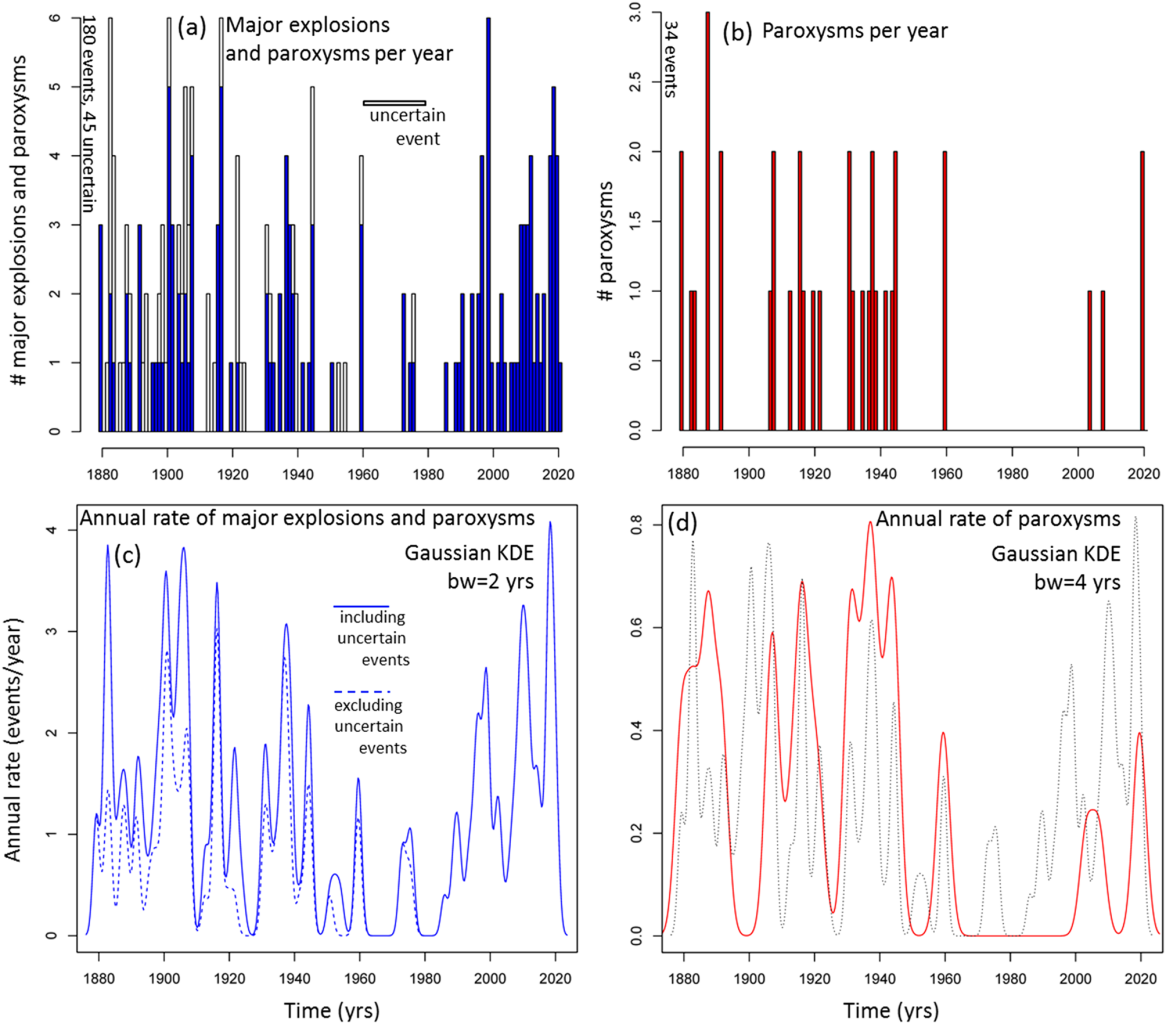
(**a**,**b**) Annual barplot and (**c**,**d**) Gaussian KDE of (**a**,**c**) the ME&P and (**b**,**d**) only the paroxysms. In (**a**) the white part of the bars marks the major explosions of uncertain characterization. In (**c**) the dashed line does not include these uncertain events. In (**d**) the dotted line is the KDE of the ME&P scaled of five times and included as a comparison.

In Fig. 3a,b we display an annual barplot and in Fig. 3c,d a Gaussian Kernel Density Estimator (KDE^42,54,126^) of the ME&P record and of the paroxysms alone. In [1879, 2020] we count 180 ME&P, of which 45 have an uncertain characterization (all major explosions), and 36 paroxysms (none is uncertain). No major explosions in [1985, 2020] are assumed uncertain. The annual barplot shows that there were [0, 6] major explosions per year, and [0, 3] paroxysms per year, from 1879 (Fig. 3a,b). The Gaussian KDE enables characterization of the temporal groups of events regardless of the annual discretization. We selected an arbitrary bandwidth 2σ = 2 years for major explosions, and 2σ = 4 years for paroxysms. However, such a choice does not significantly affect the results reported here. This plot provides a visual summary of the historic data and does not have a forecasting purpose. From the figure a decrease in the number of reported events per year is evident between 1960 and 1990. The local maxima in the number of major explosions and paroxysms are not always coincident—e.g. 1900, 1998, and 2010 are significant peaks in the major explosions record but without paroxysms (Fig. 3c,d). In Fig. 3d we count six main groups of paroxysms.

**Figure 4:**
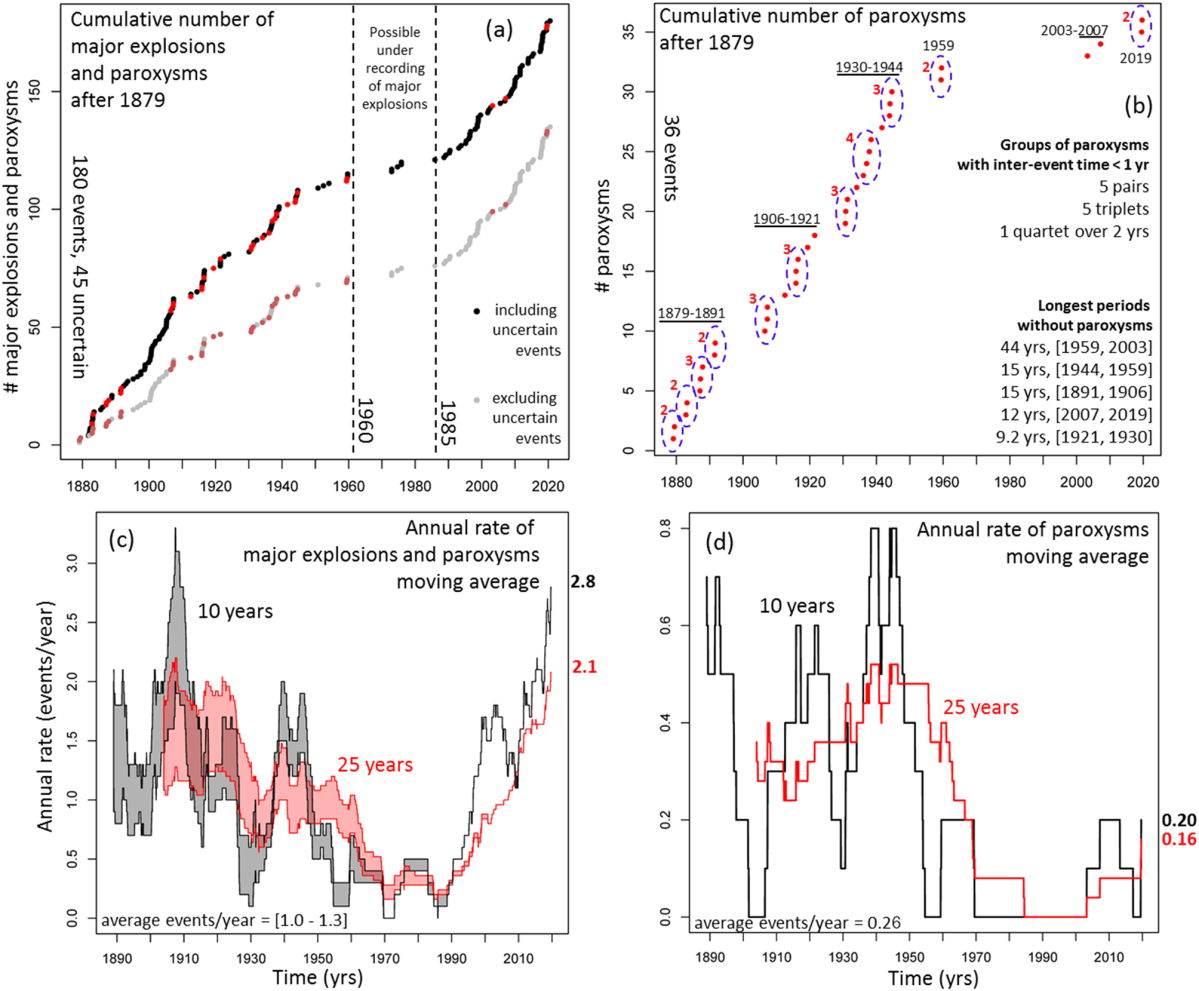
(**a**,**b**) Cumulative number after 1879 and (**c**,**d**) annual rate of (**a**,**c**) ME&P and (**b**,**d**) only paroxysms. In (**a**) the grey dots do not consider the events of uncertain characterization (all major explosions). The red dots are the paroxysms. We marked the time interval with possible under-recording issue with dashed lines. In (**b**) we circled the groups of paroxysms with less than 1-year inter-event time, and we labeled six main groups of paroxysms separated by more than 9-year inter-event time. On the right of (**b**), we listed the size of the 1-year groups, and the size of the five longest periods without paroxysms. In (**c**,**d**) the annual rate is obtained with a moving average of length 10 years (black) and 25 years (red). We display the current annual rate estimates on the right, and the average number of events/year over the whole period on the left.

In Fig. 4**a**,**b** we display the cumulative number and in Fig. 4**c**,**d** the annual rate of ME&P events and of paroxysms by themselves. We base the estimate of the annual rate on left-side first order finite differences^44^. We use left-side intervals so that the value at time t is not anticipating future information that is unavailable at that time. The current annual rate of ME&P is 2.8 events/year based on the last 10 years, and 2.1 events per year based on the last 25 years. The average rate over the last 140 years is in the range [1.0, 1.3] events/year, i.e. about half of the most recent rates. The annual rates in [1995, 2020] are close to the maximum rates observed in [1879, 1908] and higher than those in [1908, 1960] (Fig. 4c).

The current annual rate of paroxysms is 0.20 events/year based on the last 10 years, and 0.16 events per year based on the last 25 years. The average rate in the last 140 years is 0.26 events/year, i.e. 20–50% higher than the current rate. The annual rates in [1985, 2020] are 3–4 times lower than the maximum rates observed in [1879, 1960] (Fig. 4d). The temporal grouping of paroxysms is also apparent—six main groups separated by five periods, each of at least 9 year’s duration. The longest period without paroxysms was 44 years (i.e. between 1959 and 2003). On a finer scale, we observe 5 pairs, 5 triplets and 1 quartet of events separated by less than 1-year inter-event time. Only 7/36 = 19% of the events do not belong to any of these 1-year groups (Fig. 4b).

In the last 140 years 20–27% of ME&P were paroxysms, while in the last 35 years only 6.7% of the ME&P were paroxysms. This means that, on average, from 1879 to 2020 there was a paroxysm every 3–4 major explosions, but from 1985 to 2020 there was a paroxysm every 14 major explosions. We finally note that, although the maxima of major explosions are not always coincident with paroxysms, the annualized rate of paroxysms in the 15 days after any major explosion is 1.35 events/year, i.e. 5–6 times more likely than usual to have a paroxysm. In contrast, the annualized rate of paroxysms quickly decreases to 0.27 events/year within about 15–30 days after any major explosion. A table of all the inter-event times of paroxysms is available as Supporting Information S2.

## Statistical models of the inter-event time

**Figure 5:**
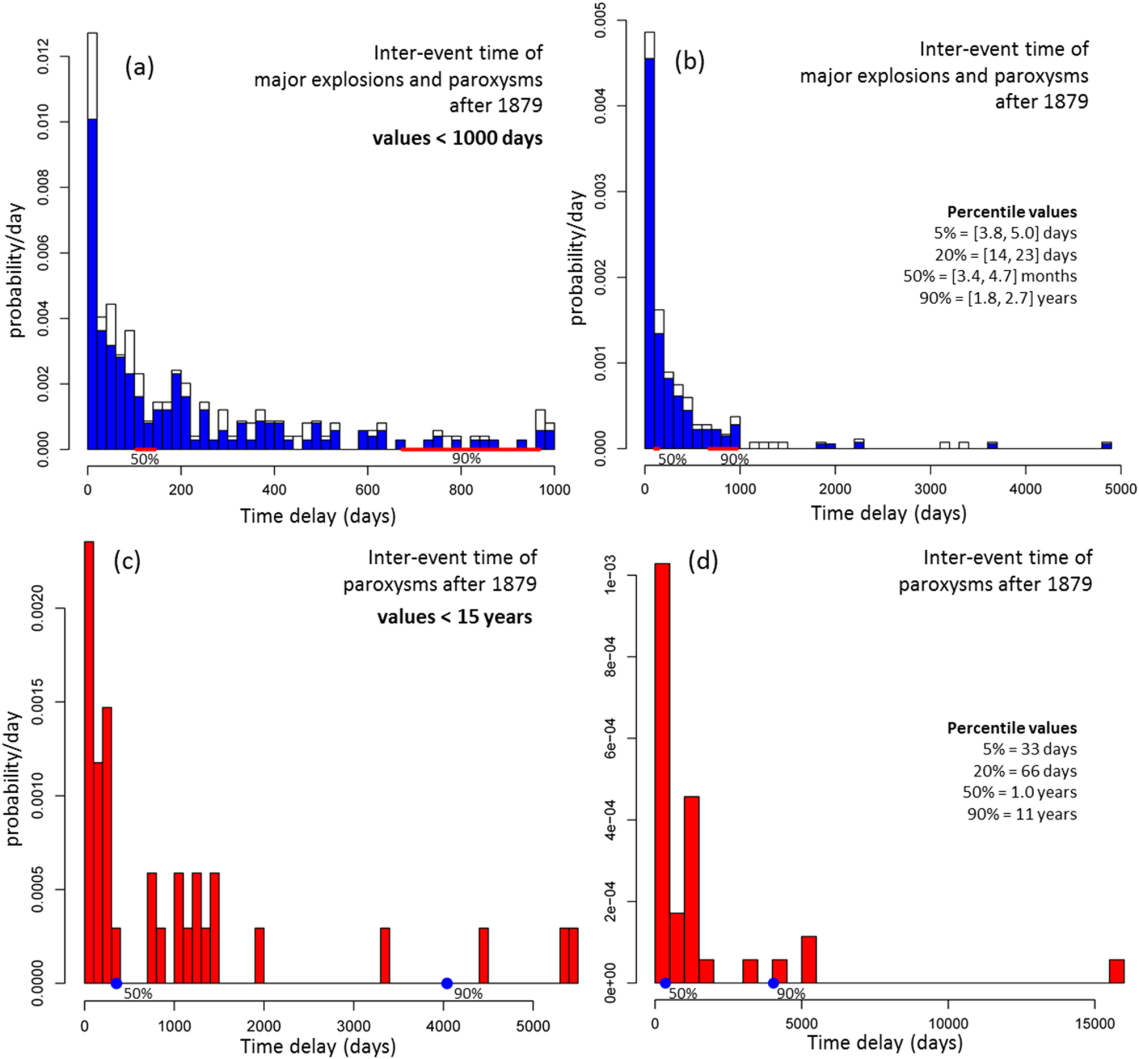
Histograms of the inter-event time of (**a**,**b**) ME&P, in blue, and (**c**,**d**) only the paroxysms, in red. We consider all the pairs of consecutive events observed after 1879. (**a**,**c**) A zoom on the values lower than 1000 days and 15 years respectively. In (**a**,**b**) the white part of the bars marks the differences due to major explosions of uncertain characterization. The red lines mark the uncertain values of 50th and 90th percentiles of the distribution. In (**c**,**d**) the blue dots mark the 50th and 90th percentiles. In (**a**,**c**) the 5th, 20th, 50th and 90th percentile values are listed.

Figure 5 displays the histograms of the inter-event time of ME&P events and of paroxysms only. These statistics describe the entire dataset post 1879, and not only the current annual rate. The figure reports the percentile values of the data. The inter-event time of ME&P events has 5th percentile value in [3.8, 5.0] days, median value in [3.4, 4.7] months, and 90th percentile in [1.8, 2.7] years (Fig. 5a,b). The inter-event time of the paroxysms has a 5th percentile value of 33 days, median value of 1.0 years, and 90th percentile of 11 years. The maximum value of 44 years is about three times larger than the second greatest value, 15 years, and represents a peculiarity in the data (see below for further discussion) (Fig. 5c,d).

### Analysis models of ME&P record and of paroxysms only, between 1879 and 2020

We develop analysis models for representing the probability distribution of the inter-event time of ME&P events and of paroxysms only. We obtained Maximum Likelihood Estimators (MLE) of exponential, Weibull, and lognormal distributions. The exponential class represents the inter-event distribution of a classical Poisson process. This is a common choice, but restrictive^27,40^. Furthermore, the short-term analysis of the explosive activity at Stromboli indicates that the system exhibits some memory^75,76^. The lognormal class produces a higher likelihood of observing extreme values, either small or very large, when compared to an exponential with the same mean value. This tends to produce clusters of events and long periods without events. The Weibull class has intermediate properties between an exponential and a lognormal^34,82^.

In addition, we introduced a simple approach that enables us to model short and long inter-event time separately. In particular, we divided the data in two subsets of the same size—inter-event times that are lower than the median value, i.e. the “body” of the data, and those greater than the median value, i.e. the upper “tail” of the data. Then, we separately calculated maximum likelihood exponential, Weibull, and lognormal functions on these subsets. Finally, we defined an integrated model as the linear combination of the maximum likelihood functions on the two subsets, with equal weights. This probability mixture can exploit significantly different properties in the body and in the tail of the data, at the cost of requiring a greater number of modeling parameters. This approach is a Markov chain with two states with equal chances, which represent the body and the tail of the data, respectively^25,31,130^.

We compared the probability classes, and the possibility of splitting the body and the tail of the data, by using the Akaike criterion, which maximizes the difference of log-likelihood and the number of modeling parameters^5,6^. In particular, the exponential class uses one parameter, the lognormal and Weibull classes use two parameters. A table of all the MLE solutions computed is available as Supporting Information S3.

**Figure 6:**
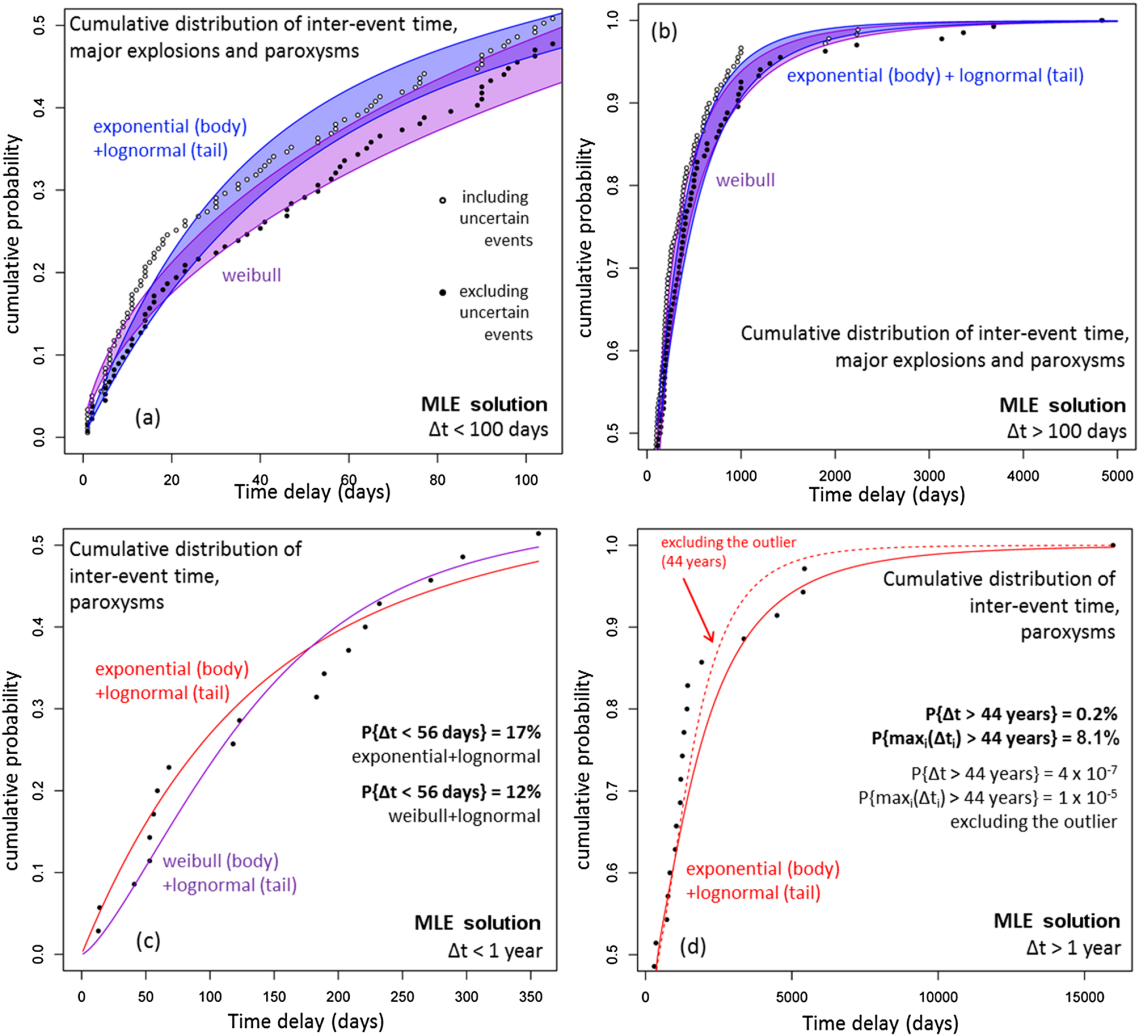
Cumulative distribution functions of the recorded inter-event time and of the best performing probability models according to the Akaike criterion^5,6^. Different colors mean different models. (**a**,**b**) ME&P and (**c**,**d**) only the paroxysms. In (**a**,**b**) the black dots do not consider the events of uncertain characterization. A colored region marks the envelope of the probability model that includes the uncertain events and the model that does not. In (**a**,**c**) we focus on the body of the distribution, i.e. Δt lower than the median value, in (**b**,**d**) on the tail, i.e. Δt greater than the median value. In (**c**) we report the probability values of an inter-event time lower than 56 days, in (**d**) the probability of an inter-event time greater than 44 years, and probability of the maximum of 35 independent inter-event times to be greater than 44 years (see text for more explanation).

Figure 6 shows the cumulative distribution functions of the inter-event times. The best modeling choices of the ME&P data are a Weibull function, or the combination of an exponential function for the body and a lognormal function for the tail of the data (Fig. 6a,b). In contrast, the best modeling choices for the paroxysms data are the combination of an exponential or a Weibull function for the body, with a lognormal function for the tail of the data (Fig. 6c,d).

We calculated that an inter-event time less than 56 days, i.e. the time between the two paroxysms in 2019 (July 3 and August 28), has a probability of 12% by assuming the exponential form, and 17% from the Weibull distribution on the body of the data, respectively (Fig. 6c). An inter-event time greater than 44 years (since 1959–2003) has a probability of 0.2%, assuming a lognormal distribution on the tail of the data. Moreover, the probability that the maximum inter-event time over 35 independent samples exceeds 44 years is 8.1% (Fig. 6d). To make a comparison, we also tested a lognormal model obtained excluding the 44-year outlier—that slightly improves the replication of the inter-event times less than 5000 days, but the probability to observe that value becomes negligible.

We remark that once a statistical model of inter-event times is selected, the simulation of a range of plausible realizations of event-times distributions becomes possible. In particular, through these simulations, and the study of their properties, it may be possible to identify improvements in the inter-event time modeling, and using any insight to better interpret the underlying volcano dynamics. Furthermore, a stochastic simulation of the Stromboli catalog, possibly accounting for epistemic uncertainties affecting the characterization of past events^40^ and the statistical uncertainty affecting the MLE^44^ solutions, would provide a basis for considering different realizations of tolerable exposure time and when these might cause operational challenges for protocols. We finally note that renewal processes, even when they use two different Markov states, imply that the volcano ‘resets’ its memory after each large explosion. More advanced approaches may include more memory of the past, for example using mechanisms of cumulative self-excitement^40,44^. Research in this direction may further improve inter-event time modeling.

### Analysis models of the ME&P record in periods [1879, 1960] and [1985, 2020]

The ME&P record is likely affected by under recording of major explosions occurred between 1960 and 1985, due to the cessation on Stromboli of military observations. Therefore, we separately analyzed the historical record in [1879, 1960] and the more recent record in [1985, 2020].

**Figure 7:**
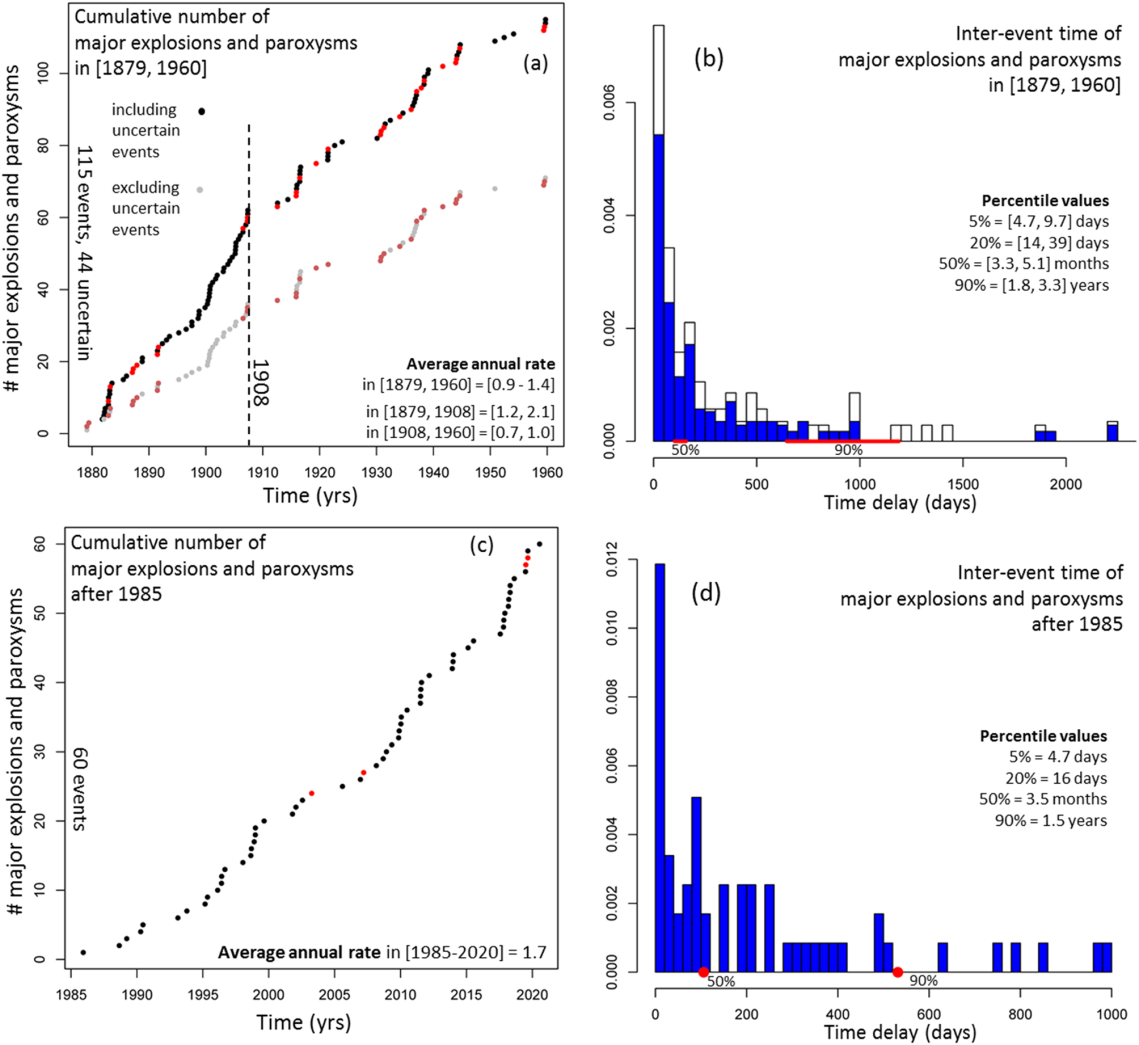
(**a**,**c**) cumulative number and (**b**,**d**) histograms of inter-event time of ME&P record (**a**,**b**) in [1879, 1960] and (**c**,**d**) in [1985, 2020]. In (**a**) the grey dots do not consider the events of uncertain characterization. The red dots are the paroxysms. We marked the year 1908 with a dashed line to highlight the average rate change. In (**b**) the white part of the bars marks the major explosions of uncertain characterization. In (**b**,**d**) the red lines mark the uncertain values of the 50th and 90th percentiles of the distribution. On the right of (**b**,**d**) we listed the 5th, 20th, 50th and 90th percentile values.

Figure 7 shows the cumulative number and the histograms of their inter-event times for the ME&P record in these two time intervals. In [1879, 1960], the average rate is in [0.9, 1.4] events/year, i.e. about half current rates. We note that the rate is not uniform, and in [1879, 1908] the average rate is in [1.2, 2.1], while in [1908, 1960] it is in [0.7, 1.0] (Fig. 7a). The inter-event time in [1879, 1960] has 5th percentile value in [4.7, 9.7] days, median value in [3.3, 5.1] months, and 90th percentile in [1.8, 3.3] years (Fig. 7b).

In [1985, 2020] the average rate is 1.7 events/year (Fig. 7c). The inter-event time observed in this recent time interval has 5th percentile value of 4.6 days, median value of 3.3 months, and 90th percentile of 1.5 years, similar to the values observed in [1879, 1960] when including the major explosions of uncertain characterization (Fig. 7d).

**Figure 8:**
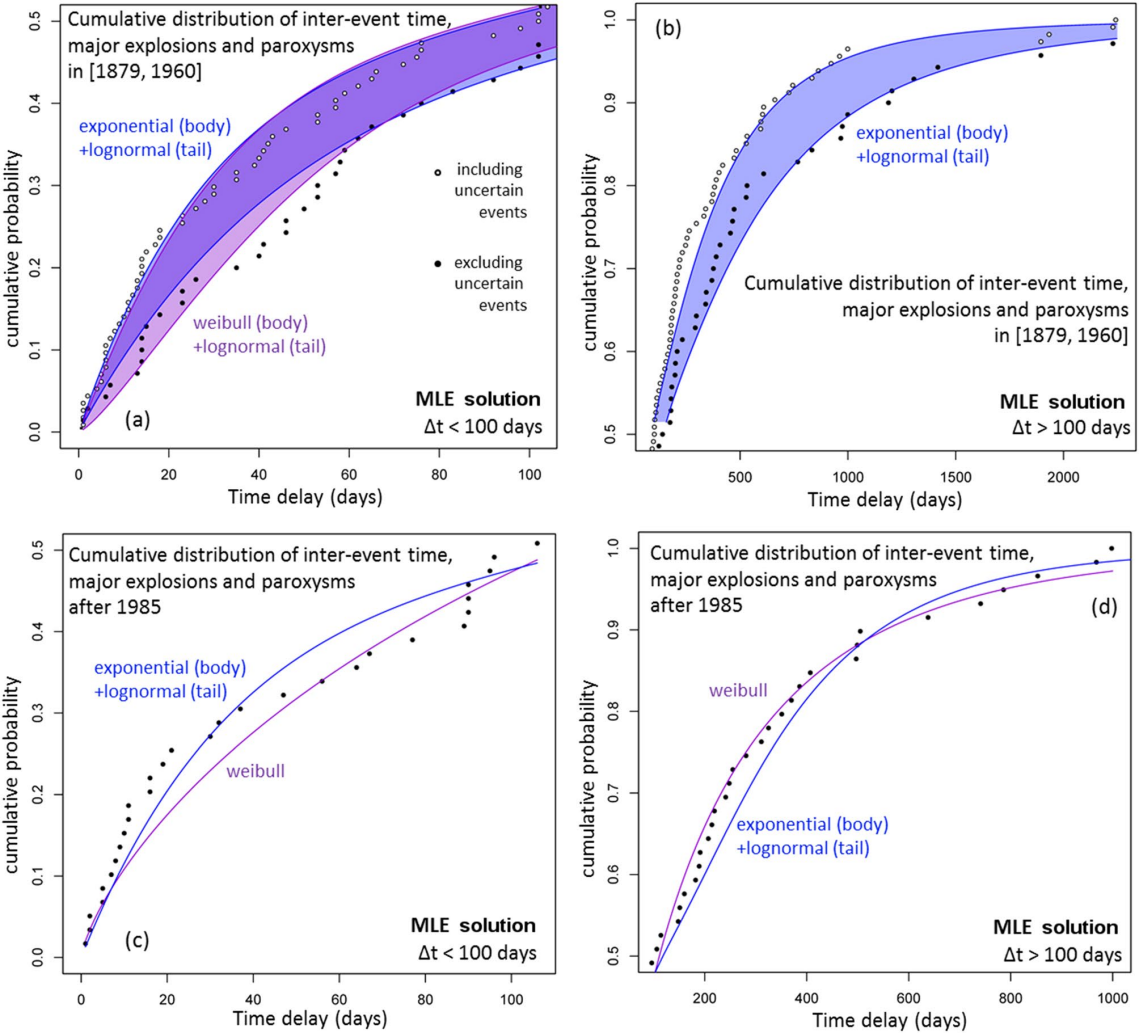
Cumulative distribution functions of the recorded inter-event time and of the best performing probability models according to the Akaike criterion. Different colors mean different models. (**a**,**b**) shows ME&P record in [1879, 1960] and (**c**,**d**) in [1985, 2020]. In (**a**,**b**) the black dots do not consider the events of uncertain characterization. A colored region marks the envelope of the probability model that includes the uncertain events and the model that does not. (**a**,**c**) focus on the body of the distribution, i.e. Δt < 100 days, (**b**,**d**) on the tail, i.e. Δt > 100 days (see text for more explanation).

Figure 8 shows the cumulative distribution functions of the inter-event times of ME&P events in [1879, 1960] and in [1985, 2020]. The best modeling choices for [1879, 1960] are the combination of an exponential or a Weibull function for the body, with a lognormal function for the tail of the data (Fig. 8a,b). For [1985, 2020], the best modeling choices are a Weibull function, and the combination of an exponential function for the body and a lognormal function for the tail of the data (Fig. 8c,d). In summary, a lognormal function represents well the tail of the distribution, even after dividing the ME&P record into two disjoint parts. A table of all the MLE solutions computed is available as Supporting Information S3.

## Temporal rates of major explosions and paroxysms conditional on the time passed from the last event

We can use our probability models of the inter-event times to provide quantitative estimates of the temporal rates of ME&P events as a function of time since the last event occurred, i.e. the hazard function^41,56,57^. If T is a discrete random time, its hazard function is P_T_(t): = P{T = t|T ≥ t}. If T is continuously distributed with probability density function f_T_, then P_T_(t): = f_T_ (t)/P{T ≥ t}. In practice, the hazard function indicates which periods have the highest or lowest chances of a new ME&P in the weeks or months after the last similar event.

**Figure 9:**
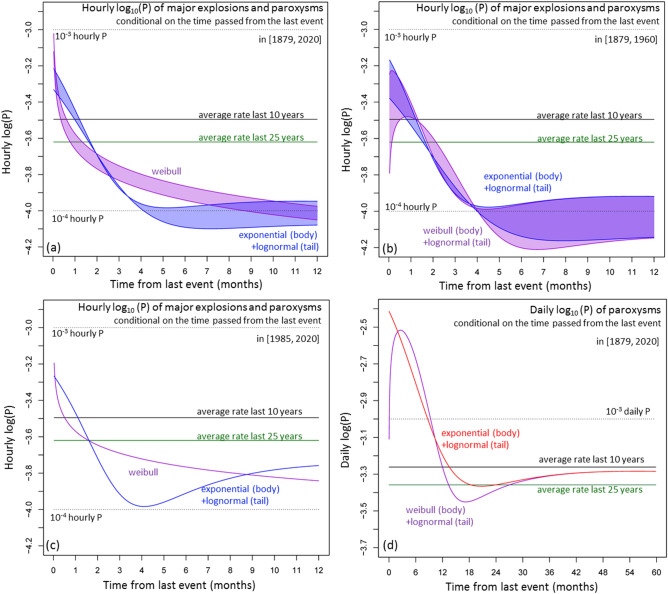
Time dependent probability rate (hazard function) of the best performing probability models according to the Akaike criterion. Different colors mean different models, reported in the labels. (**a**–**c**) Consider the ME&P record as—(**a**) in [1879, 2020], (**b**) in [1879, 1960], and (**c**) in [1985, 2020]. (**d**) Considers only the paroxysms in [1879, 2020]. In (**a**,**b**) the colored region marks the envelope of the probability model that includes the uncertain events and the model that does not. In (**a**–**c**) we report hourly rates, and dotted lines mark 10^–3^ and 10^–4^ hourly probability levels. In (**d**) we report daily rates, and a dotted line marks 10^–3^ daily probability. We report the average rates in the last 10 and 25 years for comparison.

Figure 9 shows the logarithm of the hazard function of ME&P events and of paroxysms only, i.e. the hourly or the daily log_10_(P). The hourly log_10_(P) calculated in [1879, 2020] is in [− 3.3, − 3.0] in the first week after the event, but gradually decreases to the interval [− 4.1, − 3.9] after N months without other ME&P events. N is about 3 according to the combination of an exponential and a lognormal, whereas N is about 5–8 according to a Weibull model (Fig. 9a).

In [1879, 1960] the maximum hourly log_10_(P) of a further ME&P event is in [− 3.5, − 3.2] in the first month after the event and decreases to the interval [− 4.2, − 3.9] after about 3–4 months according to each of our models. We note that the combination of a Weibull and a lognormal does not produce a maximum hazard function immediately after the event, but after 1–4 weeks. Instead, the combination of an exponential and a lognormal produces a hazard function that is decreasing initially and for the first three-to-four months, before inflection and subsequent asymptotic increase (Fig. 9b).

In [1985, 2020] the maximum hourly log_10_(P) of ME&P events is in [− 3.3, − 3.2] in the first week after the event and decreases to a log_10_(P) range [− 3.9, − 3.8], after N months. In this case, N is about 2–3 according to the combination of an exponential and a lognormal, whereas N is about 8 according to a Weibull model. Whilst the Weibull model produces a decreasing hazard function, the combination of an exponential and a lognormal produces a minimum hazard after 4 months and then gradually increases again of about 50% (Fig. 9c).

The maximum daily log_10_(P) for paroxysms as a category is in [− 2.5, − 2.4] in the first 4–6 months after the event. Then it gradually decreases to the interval [− 3.5, − 3.3] after N months, where N is about 12 according to the combination of a Weibull and a lognormal, and N is about 15 according to the combination of an exponential and a lognormal. In particular, the daily log_10_(P) slowly increases to about − 3.3 after 3 years from the event. We note that, again, the combination of a Weibull and a lognormal does not produce a maximum hazard function immediately after the event, but after 2–4 months (Fig. 9d).

Based on these computed hazard functions and under a few simplifying assumptions, we can derive order-of-magnitude estimates of the duration of the tolerable exposure time for people that climb to the summit of the volcano, e.g. volcanologists, guides, tourists. We remark that our preliminary analysis only considers the individual risk of fatality. Estimates of societal risk involve complex factors, and implications, and inter alia depend on the number of visitors in addition to the length of their exposure time. Furthermore, cost–benefit analyses including the evaluation of economic losses are outside the scope of this study and may lead to other conclusions for decision making^71,73,120^. Nevertheless, our individual risk analysis provides a first fundamental step towards carrying out a comprehensive risk assessment. Calculating individual risk, as done in this work, is particularly useful for one-off visitors if decision making is in their hands, or for individuals occasionally working in the summit area. If the decision, to access the area or not, rests not with the individual but with some legislated authority, then societal risk is also relevant. Risk mitigation measures might then include some control of the number of individuals being exposed to the risk, for protecting lives, and evaluation of potential economic losses. Such aspects are also important and should be the subject of further discussion and analyses to determine a comprehensive risk assessment.

Estimation of what constitutes a tolerable exposure time requires the determination of appropriate probability levels for different types of visitors^24^. For example, previous risk analyses^61,73,77^ chose a value of 0.1% for workers (i.e. volcanologists and guides in our context) and 0.01% for generic public (i.e. tourists in our context) as annual individual tolerable lethal risk levels. In practice the actual fatality rate for workers in even the most hazardous industries is normally well below the upper limit of a risk of death to any individual of 1 in 1000 per annum. Similarly, the fatality rate for the public who have a risk imposed on them ‘in the wider interest of society’ is usually well below 1 in 10,000 per annum. Hence, other values could be chosen, and different thresholds would linearly change the tolerable time estimates.

A threshold probability divided by the current level of the hazard function approximates, under the assumption of vulnerability equal to one, the tolerable exposure time. Of course, this simplifying approximation is valid when the hazard function does not change significantly during exposure time. Otherwise, the integral of the hazard function over the time of exposure, i.e. the cumulative hazard function, provides the total probability, and numerical inversion is required to compute the tolerable exposure time^56,57^. Consequently, in this initial study for Stromboli, we assume that only the length of time determines the exposure, i.e. the spatial component does not affect the risk level for an individual. In other words, this assumption is only valid in the summit areas of the volcano that are dramatically affected by ME&P events, but it is not true in general. Given these two main simplifying assumptions, the tolerable exposure times enumerated here should be considered as first indicative estimates of the order-of-magnitude of an acceptable exposure time for individuals at the top of the volcano.

In future analyses, we plan to introduce in the assessment the spatial component of risk exposure, for example by considering the maximum range of ballistic projectiles, their radial direction, and the spatial density of the projectiles^46,65,66^ or the volume, direction and mechanisms of propagation of pyroclastic density currents and hot avalanches^63,121,127,131^. Similarly, specific vulnerability functions should be introduced for the main hazardous actions considered^29,128^.

**Figure 10:**
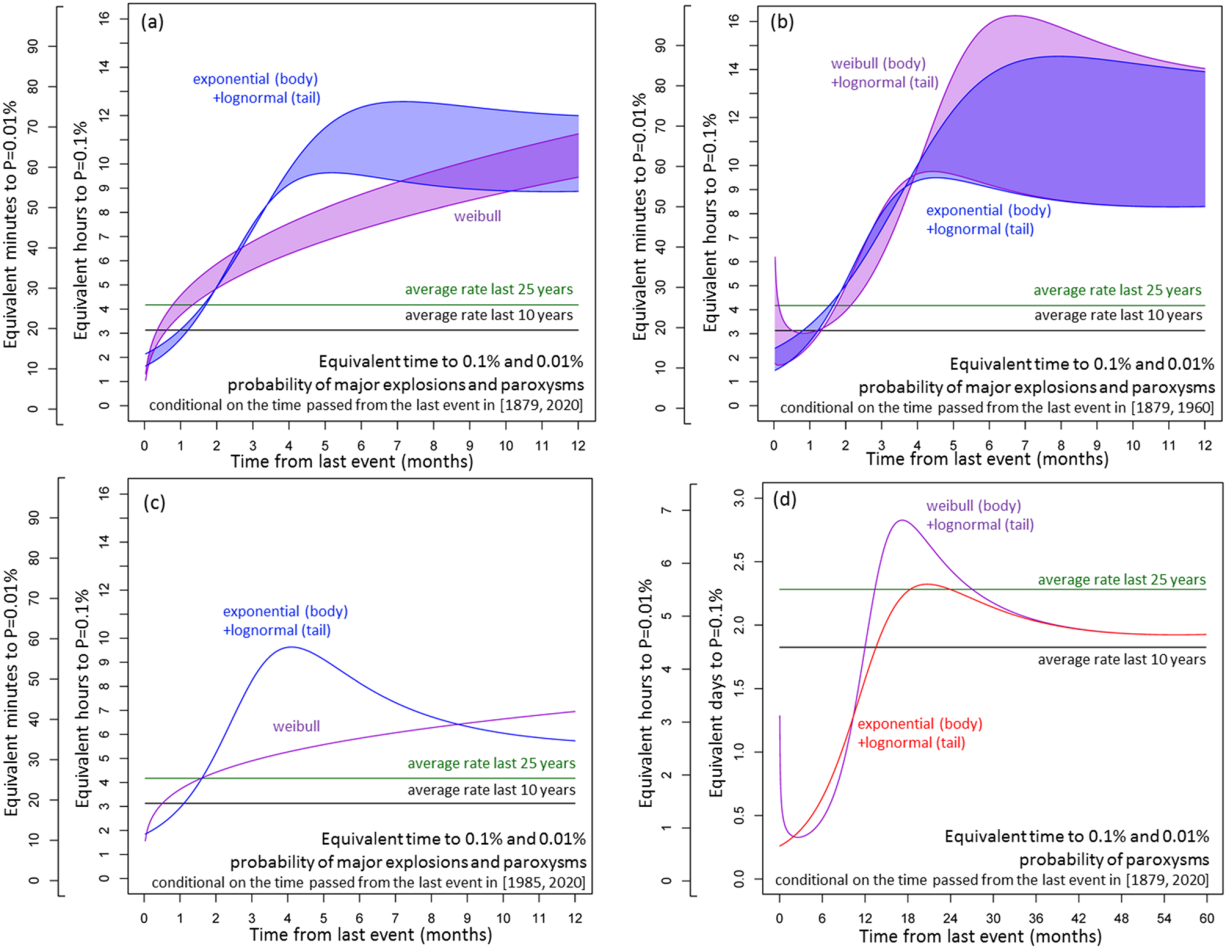
Equivalent time to 0.1% and 0.01% probability levels, conditional on the time passed from the last event. Different colors mean different models, reported in the labels. Different time scales consider different probability levels. In (**a**–**c**) we consider the ME&P record as—(**a**) in [1879, 2020], (**b**) in [1879, 1960], and (**c**) in [1985, 2020]. In (**d**) we consider only the paroxysms in [1879, 2020]. In (**a**,**b**) the colored region marks the envelope of the probability model that includes the uncertain events and the model that does not. We also report the results obtained by using the average rates in the last 10 and 25 years for comparison (see text for more explanation).

Figure 10 shows the equivalent time to occurrence of an ME&P event or a paroxysm with probability P, where P = 0.1% or P = 0.01%, conditional on the time elapsed since the last event. All the graphs are proportional to the inverse of the associated hazard function, and the chosen probability level P is a scaling factor of the equivalent time. The results concerning ME&P events show a minimum equivalent time in the first month after an event, and then it gradually increases until 3–7 months after the event (Fig. 10a–c).

The results concerning the paroxysms show a minimum equivalent time in the first 4–6 months after the event, and then it increases until 15–18 months after the event. Then the equivalent time decreases again until about 36 months after the event (Fig. 10d). Table 1 reports our estimates after 1, 3, 6 and 12 months from the last ME&P event, and after 2, 6, 12, and 24 months after the last paroxysm.

**Table 1:** Equivalent time to 0.1% and 0.01% probability levels, conditional on the time passed from the last event.

Time from last event	P = 0.1%		P = 0.01%		Time from last event	P = 0.1%		P = 0.01%	
	Exponential + lognormal	Weibull	Exponential + lognormal	Weibull		Exponential + lognormal	Weibull + lognormal	Exponential + lognormal	Weibull + lognormal
**(a) Equivalent time to P probability of major explosions and paroxysms conditional on the time passed from last event, in [1879, 2020]**					**(b) Equivalent time to P probability of major explosions and paroxysms conditional on the time passed from last event, in [1879, 1960]**				
1 month	[2.7, 3.1] h	[3.7, 4.5] h	[16, 19] min.	[22, 27] min.	1 month	[2.6, 3.3] h	[2.5, 3.0] h	[16, 20] min.	[15, 18] min.
3 months	[7.3, 7.5] h	[5.6, 6.8] h	[44, 45] min.	[34, 41] min.	3 months	[7.3, 8.0] h	[8.1, 6.2] h	[44, 48] min.	[37, 48] min.
6 months	[9.5, 12] h	[7.3, 8.7] h	[57, 74] min.	[44, 52] min.	6 months	[9.1, 14] h	[9.2, 16] h	[55, 83] min.	[55, 95] min.
12 months	[8.9, 12] h	[9.4, 11] h	[53, 72] min.	[57, 67] min.	12 months	[8.3, 14] h	[8.3, 14] h	[50, 83] min.	[50, 84] min.
**(c) Equivalent time to P probability of major explosions and paroxysms conditional on the time passed from last event, in [1985, 2020]**					**(d) Equivalent time to P probability of paroxysms conditional on the time passed from last event, in [1879, 2020]**				
1 month	2.9 h	3.7 h	17 min	22 min	2 months	0.34 days	0.33 days	0.81 h	0.80 h
3 months	8.2 h	4.9 h	49 min	29 min	6 months	0.64 days	0.47 days	1.5 h	1.1 h
6 months	8.1 h	5.8 h	49 min	35 min	12 months	1.6 days	1.8 days	3.8 h	4.4 h
12 months	5.7 h	6.9 h	34 min	42 min	24 months	2.3 days	2.4 days	5.5 h	5.9 h

In (a-c) we consider the ME&P record as—(a) in [1879, 2020], (b) in [1879, 1960], and (c) in [1985, 2020]. In (d) we consider only the paroxysms in [1879, 2020].

## Conclusions

From a detailed review of the scientific literature and sources of the last 140 years, we have constructed a new historical catalog of major explosions and paroxysms at Stromboli volcano. Then, we modeled the inter-event time data and we estimated temporal rates of major explosions and paroxysms in this catalog. A model combining exponential and lognormal functions in a two-state Markov chain works well with all the datasets. The model associates significant probabilities of occurrence to both the short inter-event time observed in the summer of 2019, and to the 44-year outlier between 1959 and 2003.

We quantified the variable temporal rate of major explosions and paroxysms in the eruptive record as a function of time elapsed since the last event occurred, i.e. the hazard function. We found that hazard levels are more elevated in the weeks following a major explosion or paroxysm and that these gradually decrease afterwards. We also quantified the main sources of uncertainty affecting the historical catalog, i.e. major explosions with uncertain characterization or properties, and possible under recording issues in the period [1960, 1985] due to the cessation on Stromboli of military observations. The results relying on the ME&P data recorded before 1960 are presented as an uncertainty range defined by two values, this meaning that one estimate includes all the uncertain events, while the other estimate excludes all of them.

In summary, the main results of the study include:

- There is a 50% probability that a major explosion or a paroxysm follows the previous one after less than [3.4, 4.7] months, and 20% probability in less than [14, 23] days. Based on the data in [1985, 2020], these estimates are 3.5 months and 16 days, respectively. In [1985, 2020] the inter-event time of ME&P events is significantly similar to [1879, 1908]. The annual rate of ME&P events in the last 10 years, 2.8 events/year, is close to the maximum observed in the first two decades of the 20th century, i.e. 3.2 events/year. The average annual rate in the last 140 years is in [1.0, 1.3] events/year. Thus, Stromboli is currently experiencing one of the most active periods of its recent history.
- There is a 50% probability that a paroxysm follows the previous one in less than one year, and 20% probability in less than 66 days. After 1879, there have been 36 paroxysms: 7 occurred as single events, 5 times the eruptions came as a pair with less than 1-year inter-event time, 5 times as a triplet, and once as a quartet. Based on these findings, there was a probability of about 55% (i.e. 6/11) that the two paroxysms of 2019 would be followed by a third paroxysm in less than one year. The annual rate of the paroxysms in the last 10 years, 0.20 events/year, is about a quarter of the maximum registered in the first decades of the 20th century, i.e. 0.80 events/year. The average annual rate in the last 140 years is 0.26 events/year. On average, from 1879 to 2020 there was a paroxysm every 3–4 major explosions, but from 1985 to 2020 there was a paroxysm every 14 major explosions. Even though the local maxima of major explosions are not always coincident with paroxysms, the annual rate of the paroxysms in the 15 days after any major explosion is 1.35 events/year, i.e. 5–6 times greater than the average rate, and then decreases rapidly.
- The hourly hazard function of ME&P events in [1879, 2020] is in the probability range [0.05%, 0.1%] in the first week after the event, but gradually decreases by 5–10 times in 3–8 months, depending on the model. In [1985, 2020] the pattern is similar, but the hazard function starts at [0.05%, 0.06%] and then decreases by about five times; according to the combination of an exponential and a lognormal function the hazard reaches the minimum after 4 months and then gradually increases again of about 50%. In the period [1879, 1960] the hazard takes 3–4 months to decrease to about 0.01%, in either of our models.
- The daily hazard function of paroxysms in [1879, 2020] is [0.3%, 0.4%] in the first 4–6 months after the event, and decreases by about ten times in 12–15 months, again depending on the model. The combination of exponential and lognormal functions shows a maximum hazard function immediately after the last paroxysm while, for the combination of Weibull and lognormal distributions, that maximum is delayed by about two to four months.
- Using data from the whole catalog period [1879, 2020], for an ME&P event the equivalent exposure times which correspond to a 0.1%, i.e. 10^–3^ risk of fatality (a possible maximum annualized risk threshold assumed for workers in previous risk analyses^73^), are about 3–4 h if it is 1 month following the event, 7–12 h if it is 6 months later, and 9–12 h if 12 months have elapsed since the last event. For members of the general public, the equivalent exposure times—corresponding to a 0.01%, i.e. 10^–4^ risk of fatality (a possible maximum annualized threshold assumed in previous risk analyses^73^), are about ten times shorter. In other words, a member of the public would exceed a tolerable risk level by being on the volcano summit areas affected by ME&P events for only about 20 min, one month after an ME&P event. Between 1985 and 2020, the risk exposure pattern as a function elapsed time since an ME&P event, is similar but, because of the increased average rate of occurrence of recent activity, the tolerable exposure time after 6 and after 12 months are ca. 30% and 50% shorter, respectively.
- Over the period [1879, 2020], for paroxysms the equivalent times with 0.1% risk probability, following an event, are: 8 h exposure 2 months later, ca. 1.5 days after 12 months, and ca. 2.5 days 24 months after the last paroxysm. The equivalent times to 0.01% probability are again ten times shorter.
- As clarified above, the equivalent time estimates are intended to serve simply as illustrative of the form of risk analysis made possible using the hazard functions we have adopted. We note that the described exposure times are valid in the summit areas of the volcano that would be affected on every occurrence of ME&P events. Thus, the assessment of a spatial component of risk exposure is a very important aspect of future research. In addition, we did not use a vulnerability function to relate risk of fatality to hazard probability^29,128^. The assumption here is that the occurrence of an eruption entails certain death. However, a case can be made that the actual probability of death in larger eruptions is quite limited because risk will decrease with distance from the craters, and there will be some chance of surviving the hazards.

The temporal analysis detailed here represents crucial input information for the development of quantitative hazard assessments due to the various hazardous phenomena, such as ballistic projectiles^7,46,62,66^, pyroclastic density currents^11,43,92^, hot avalanches^30,64,93,122^, and tsunami^67,80,99,119^. Moreover, these findings can inform a better understanding of past eruption crises on Stromboli through quantitative counterfactual analysis, i.e. the estimation of the likelihood of what actually happened and what could have happened^26,132^. Our methodology is transferable to other volcanoes and, doubtless, can be extended in its analytical scope. For instance, our approach provides a basis for setting up base rate hazard priors that can be easily updated with real-time monitoring data^45,68,69,111,112,114^. Ignoring the base rate is a well-known fallacy that compromises forecasting and diagnostic reliability in many areas of science and medicine^78^.

## Supplementary information

Supplementary Information 1

Supplementary Information 2

Supplementary Information 3
