# Supplementary material for: Major explosions and paroxysms at Stromboli (Italy): a new historical catalog and temporal models of occurrence with uncertainty quantification
Source: Sci Rep. 2020 Oct 15;10:17357. doi: 10.1038/s41598-020-74301-8 (PMC7566485; doi:10.1038/s41598-020-74301-8)
Supplement: Supplementary file 1 — Supplementary Information 1 [file 41598_2020_74301_MOESM1_ESM.docx]

**Table S1, Supporting Information to:**

**Major explosions and paroxysms at Stromboli (Italy): a new historical catalog and temporal models of occurrence with uncertainty quantification**

Andrea Bevilacqua^(1)^, Antonella Bertagnini^(1)^, Massimo Pompilio^(1)^, Patrizia Landi^(1)^, Paola Del Carlo^(1)^, Alessio Di Roberto^(1)^, Willy Aspinall^(2)^, Augusto Neri^(1)^

^1^ Istituto Nazionale di Geofisica e Vulcanologia, Sezione di Pisa, Pisa, Italy

^2^University of Bristol, School of Earth Sciences, Bristol, United Kingdom.

Corresponding author: andrea.bevilacqua@ingv.it.

LEGEND
X = Major explosion
XX = Paroxysm
X? = Uncertain major explosion

| Year | Month | Day | Type |
| --- | --- | --- | --- |
| 1879 | 2 | 4 | X |
| 1879 | 2 | 5 | XX |
| 1879 | 6 | 8 | XX |
| 1881 | 10 | 18 | X? |
| 1882 | 1 | 30 | X |
| 1882 | 3 | 13 | X? |
| 1882 | 4 | 25 | X? |
| 1882 | 11 | 17 | X? |
| 1882 | 11 | 18 | XX |
| 1882 | 11 | 24 | X? |
| 1883 | 2 | 8 | X? |
| 1883 | 2 | 9 | X |
| 1883 | 3 | 16 | XX |
| 1883 | 7 | 3 | X? |
| 1885 | 6 | 28 | X? |
| 1886 | 1 | 22 | X? |
| 1887 | 1 | 31 | XX |
| 1887 | 3 | 31 | XX |
| 1887 | 11 | 18 | XX |
| 1888 | 10 | 23 | X |
| 1888 | 11 | 5 | X? |
| 1891 | 6 | 24 | XX |
| 1891 | 6 | 30 | X |
| 1891 | 8 | 31 | XX |
| 1892 | 5 | 11 | X? |
| 1893 | 1 | 30 | X? |
| 1893 | 8 | 11 | X? |
| 1895 | 3 | 29 | X |
| 1896 | 7 | 13 | X |
| 1897 | 7 | 17 | X |
| 1897 | 7 | 18 | X? |
| 1898 | 8 | 24 | X? |
| 1898 | 10 | 20 | X? |
| 1898 | 10 | 25 | X |
| 1899 | 11 | 10 | X? |
| 1900 | 4 | 10 | X |
| 1900 | 5 | 20 | X |
| 1900 | 8 | 4 | X |
| 1900 | 8 | 22 | X |
| 1900 | 9 | 19 | X? |
| 1900 | 10 | 19 | X |
| 1901 | 3 | 8 | X |
| 1901 | 9 | 22 | X |
| 1901 | 12 | 29 | X |
| 1903 | 1 | 22 | X |
| 1903 | 3 | 9 | X |
| 1903 | 11 | 11 | X? |
| 1904 | 3 | 19 | X |
| 1904 | 8 | 22 | X? |
| 1905 | 2 | 25 | X |
| 1905 | 3 | 15 | X? |
| 1905 | 4 | 7 | X? |
| 1905 | 4 | 16 | X |
| 1905 | 9 | 27 | X? |
| 1906 | 1 | 19 | X? |
| 1906 | 7 | 11 | X? |
| 1906 | 7 | 15 | XX |
| 1907 | 1 | 11 | X |
| 1907 | 4 | 13 | XX |
| 1907 | 4 | 27 | XX |
| 1907 | 5 | 14 | X? |
| 1907 | 5 | 20 | X |
| 1912 | 7 | 27 | XX |
| 1912 | 8 | 7 | X? |
| 1914 | 5 | 30 | X? |
| 1915 | 11 | 13 | XX |
| 1915 | 11 | 26 | XX |
| 1915 | 12 | 10 | X |
| 1916 | 1 | 5 | X |
| 1916 | 7 | 2 | X |
| 1916 | 7 | 4 | XX |
| 1916 | 7 | 11 | X |
| 1916 | 7 | 22 | X? |
| 1916 | 8 | 26 | X |
| 1919 | 5 | 22 | XX |
| 1921 | 6 | 4 | X? |
| 1921 | 6 | 16 | X? |
| 1921 | 6 | 22 | X? |
| 1921 | 6 | 27 | XX |
| 1922 | 8 | 20 | X? |
| 1923 | 12 | 15 | X? |
| 1930 | 2 | 3 | X? |
| 1930 | 9 | 11 | XX |
| 1930 | 10 | 22 | XX |
| 1931 | 4 | 23 | XX |
| 1931 | 7 | 7 | X? |
| 1932 | 6 | 3 | X |
| 1934 | 2 | 2 | XX |
| 1934 | 8 | 21 | X |
| 1936 | 1 | 31 | XX |
| 1936 | 5 | 12 | X |
| 1936 | 8 | 22 | X |
| 1936 | 10 | 26 | X |
| 1937 | 1 | 6 | X |
| 1937 | 1 | 21 | XX |
| 1937 | 11 | 14 | XX |
| 1938 | 5 | 8 | X |
| 1938 | 5 | 22 | XX |
| 1938 | 6 | 1 | X? |
| 1939 | 1 | 4 | X? |
| 1939 | 2 | 12 | X? |
| 1941 | 8 | 22 | XX |
| 1943 | 12 | 3 | XX |
| 1944 | 1 | 25 | XX |
| 1944 | 2 | 24 | X? |
| 1944 | 6 | 15 | X? |
| 1944 | 8 | 20 | XX |
| 1944 | 9 | 12 | X |
| 1950 | 10 | 20 | X |
| 1952 | 6 | 7 | X? |
| 1954 | 2 | 1 | X? |
| 1959 | 5 | 19 | XX |
| 1959 | 7 | 11 | XX |
| 1959 | 9 | 6 | X |
| 1959 | 9 | 14 | X? |
| 1972 | 12 | 5 | X |
| 1972 | 12 | 10 | X |
| 1974 | 9 | 9 | X |
| 1975 | 11 | 4 | X? |
| 1975 | 11 | 5 | X |
| 1985 | 12 | 6 | X |
| 1988 | 8 | 30 | X |
| 1989 | 3 | 25 | X |
| 1990 | 4 | 15 | X |
| 1990 | 6 | 18 | X |
| 1993 | 2 | 10 | X |
| 1993 | 10 | 16 | X |
| 1995 | 3 | 5 | X |
| 1995 | 5 | 11 | X |
| 1996 | 2 | 16 | X |
| 1996 | 6 | 1 | X |
| 1996 | 6 | 6 | X |
| 1996 | 9 | 4 | X |
| 1998 | 1 | 16 | X |
| 1998 | 8 | 23 | X |
| 1998 | 9 | 8 | X |
| 1998 | 11 | 24 | X |
| 1998 | 12 | 26 | X |
| 1998 | 12 | 28 | X |
| 1999 | 8 | 26 | X |
| 2001 | 10 | 20 | X |
| 2002 | 1 | 23 | X |
| 2002 | 7 | 24 | X |
| 2003 | 4 | 5 | XX |
| 2005 | 8 | 5 | X |
| 2006 | 12 | 15 | X |
| 2007 | 3 | 15 | XX |
| 2008 | 2 | 29 | X |
| 2008 | 9 | 7 | X |
| 2008 | 12 | 6 | X |
| 2009 | 5 | 3 | X |
| 2009 | 11 | 8 | X |
| 2009 | 11 | 24 | X |
| 2010 | 1 | 10 | X |
| 2010 | 1 | 21 | X |
| 2010 | 6 | 30 | X |
| 2011 | 7 | 5 | X |
| 2011 | 7 | 10 | X |
| 2011 | 7 | 17 | X |
| 2011 | 8 | 5 | X |
| 2012 | 3 | 6 | X |
| 2013 | 12 | 4 | X |
| 2013 | 12 | 25 | X |
| 2014 | 1 | 4 | X |
| 2015 | 2 | 15 | X |
| 2015 | 7 | 16 | X |
| 2017 | 7 | 26 | X |
| 2017 | 10 | 23 | X |
| 2017 | 11 | 1 | X |
| 2017 | 12 | 1 | X |
| 2018 | 3 | 7 | X |
| 2018 | 3 | 18 | X |
| 2018 | 4 | 24 | X |
| 2018 | 4 | 26 | X |
| 2018 | 8 | 18 | X |
| 2019 | 6 | 25 | X |
| 2019 | 7 | 3 | XX |
| 2019 | 8 | 28 | XX |
| 2019 | 8 | 29 | X |
| 2020 | 7 | 19 | X |
