# Supplementary material for: Major explosions and paroxysms at Stromboli (Italy): a new historical catalog and temporal models of occurrence with uncertainty quantification
Source: Sci Rep. 2020 Oct 15;10:17357. doi: 10.1038/s41598-020-74301-8 (PMC7566485; doi:10.1038/s41598-020-74301-8)
Supplement: Supplementary file 2 — Supplementary Information 2 [file 41598_2020_74301_MOESM2_ESM.docx]

**Table S2, Supporting Information to:**

**Major explosions and paroxysms at Stromboli (Italy): a new historical catalog and temporal models of occurrence with uncertainty quantification**

Andrea Bevilacqua^(1)^, Antonella Bertagnini^(1)^, Massimo Pompilio^(1)^, Patrizia Landi^(1)^, Paola Del Carlo^(1)^, Alessio Di Roberto^(1)^, Willy Aspinall^(2)^, Augusto Neri^(1)^

^1^ Istituto Nazionale di Geofisica e Vulcanologia, Sezione di Pisa, Pisa, Italy

^2^University of Bristol, School of Earth Sciences, Bristol, United Kingdom.

Corresponding author: andrea.bevilacqua@ingv.it.

| year | month | day | inter-event time |  | year | month | day | inter-event time |
| --- | --- | --- | --- | --- | --- | --- | --- | --- |
| 1879 | 2 | 5 | 123 days |  |  |  |  | **9.2 years** |
| 1879 | 6 | 8 |  |  | 1930 | 9 | 11 | 41 days  183 days |
|  |  |  | **3.4 years** |  | 1930 | 10 | 22 |  |
| 1882 | 11 | 18 | 118 days |  | 1931 | 4 | 23 |  |
| 1883 | 3 | 16 |  |  |  |  |  | **2.8 years** |
|  |  |  | **3.9 years** |  | 1934 | 2 | 2 | - |
| 1887 | 1 | 31 | 59 days  232 days |  |  |  |  | **2.0 years** |
| 1887 | 3 | 31 |  |  | 1936 | 1 | 31 | 356 days  297 days  189 days |
| 1887 | 11 | 18 |  |  | 1937 | 1 | 21 |  |
|  |  |  | **3.6 years** |  | 1937 | 11 | 14 |  |
| 1891 | 6 | 24 | 68 days |  | 1938 | 5 | 22 |  |
| 1891 | 8 | 31 |  |  |  |  |  | **3.3 years** |
|  |  |  | **15 years** |  | 1941 | 8 | 22 | - |
| 1906 | 7 | 15 | 272 days  14 days |  |  |  |  | **2.3 years** |
| 1907 | 4 | 13 |  |  | 1943 | 12 | 3 | 53 days  208 days |
| 1907 | 4 | 27 |  |  | 1944 | 1 | 25 |  |
|  |  |  | **5.3 years** |  | 1944 | 8 | 20 |  |
| 1912 | 7 | 27 | - |  |  |  |  | **15 years** |
|  |  |  | **3.3 years** |  | 1959 | 5 | 19 | 53 days |
| 1915 | 11 | 13 | 13 days  221 days |  | 1959 | 7 | 11 |  |
| 1915 | 11 | 26 |  |  |  |  |  | **44 years** |
| 1916 | 7 | 4 |  |  | 2003 | 4 | 5 | - |
|  |  |  | **2.9 years** |  |  |  |  | **3.9 years** |
| 1919 | 5 | 22 | - |  | 2007 | 3 | 15 | - |
|  |  |  | **2.1 years** |  |  |  |  | **12.3 years** |
| 1921 | 6 | 27 | - |  | 2019 | 7 | 3 | 56 days |
|  |  |  | **9.2 years** |  | 2019 | 8 | 28 |  |
