# Supplementary material for: Major explosions and paroxysms at Stromboli (Italy): a new historical catalog and temporal models of occurrence with uncertainty quantification
Source: Sci Rep. 2020 Oct 15;10:17357. doi: 10.1038/s41598-020-74301-8 (PMC7566485; doi:10.1038/s41598-020-74301-8)
Supplement: Supplementary file 3 — Supplementary Information 3 [file 41598_2020_74301_MOESM3_ESM.docx]

**Table S3, Supporting Information to:**

**Major explosions and paroxysms at Stromboli (Italy): a new historical catalog and temporal models of occurrence with uncertainty quantification**

Andrea Bevilacqua^(1)^, Antonella Bertagnini^(1)^, Massimo Pompilio^(1)^, Patrizia Landi^(1)^, Paola Del Carlo^(1)^, Alessio Di Roberto^(1)^, Willy Aspinall^(2)^, Augusto Neri^(1)^

^1^ Istituto Nazionale di Geofisica e Vulcanologia, Sezione di Pisa, Pisa, Italy

^2^University of Bristol, School of Earth Sciences, Bristol, United Kingdom.

Corresponding author: andrea.bevilacqua@ingv.it.

| **Model parameters of the ME&P inter-event time in [1879, 2020]** | | | | |
| --- | --- | --- | --- | --- |
| (a) | **exponential +lognormal** | | **Weibull** | |
|  | **exponential** rate | **lognormal** mean(log)  sd(log) | shape  scale | |
|  |  |  |  |  |
| parameters | [0.0226, 0.0296] | [5.89, 6.16]  [0.816, 0.840] | [0.628, 0.637] [196, 266] | |
|  |  |  |  |  |
|  |  |  |  |  |
| **Model parameters of the ME&P inter-event time in [1879, 1960]** | | | | |
| (b) | **exponential +lognormal** | | **Weibull +lognormal** | |
|  | **exponential** rate | **lognormal** mean(log)  sd(log) | **Weibull** shape  scale | **lognormal** mean(log)  sd(log) |
|  |  |  |  |  |
| parameters | [0.0202, 0.0331] | [5.84, 6.30]  [0.801, 0.838] | [1.06, 1.29] [30.9, 53.1] | [5.84, 6.30]  [0.801, 0.838] |
|  |  |  |  |  |
|  |  |  |  |  |
| **Model parameters of the ME&P inter-event time in [1985, 2020]** | | | | |
| (c) | **exponential +lognormal** | | **Weibull** | |
|  | **exponential** rate | **lognormal** mean(log)  sd(log) | shape  scale | |
|  |  |  |  |  |
| parameters | 0.0263 | 5.79  0.600 | 0.747  181 | |
|  |  |  |  |  |
|  |  |  |  |  |
| **Model parameters of the paroxysms inter-event time in [1879, 2020]** | | | | |
| (d) | **exponential +lognormal** | | **Weibull +lognormal** | |
|  | **exponential** rate | **lognormal** mean(log)  sd(log) | **Weibull** shape  scale | **lognormal** mean(log)  sd(log) |
|  |  |  |  |  |
| parameters | 0.00773 | 7.52 0.834 | 1.37 141 | 7.52 0.834 |
